# Women in the Workplace: Promoting Healthy Lifestyles and Mitigating Weight Gain during the Preconception, Pregnancy, and Postpartum Periods

**DOI:** 10.3390/ijerph17030821

**Published:** 2020-01-28

**Authors:** Seonad K. Madden, Helen Skouteris, Cate Bailey, Andrew P. Hills, Kiran D. K. Ahuja, Briony Hill

**Affiliations:** 1 School of Health Sciences, College of Health and Medicine, University of Tasmania, Newnham 7248, Australia; 2 Monash Centre for Health Research and Implementation, School of Public Health and Preventive Medicine, Monash University, Level 1, 43-51 Kanooka Grove, Clayton, Victoria 3168, Australia

**Keywords:** pregnancy, preconception, postpartum, women’s health, obesity, weight management, workplace

## Abstract

Overweight and obesity before, during, and after pregnancy are associated with adverse outcomes for mothers and their offspring. Workplaces have been identified as important settings for improving health and wellbeing. However, the value of workplace interventions for women across the reproductive life stages has yet to be realized. This paper aims to explore the potential of workplaces to facilitate healthy lifestyle behaviors, prevent further weight gain, and devise tailored interventions for working women, specifically during the preconception, pregnancy, and postpartum periods. Workplaces can be used to engage women, including preconception women, who are detached from clinical settings. Potential benefits of workplace health promotion for women and employers include improved employee wellbeing, productivity, and corporate competitiveness. However, workplaces also need to overcome implementation barriers such as activity scheduling and availability. A systems approach may address these barriers. Consequently, designing and implementing workplace health promotion interventions to meet the specific needs of working women of reproductive age will necessitate collaboration with a range of key stakeholders across all stages of intervention design. Given that these women make up a considerable proportion of the workforce, workplaces can help optimize the health status of employees and prevent excess weight gain during the preconception, pregnancy, and postpartum periods.

## 1. Introduction

Two-thirds of women from high-income countries have overweight or obesity [1], which are important risk factors for many chronic diseases, including cancer, cardiovascular disease, chronic kidney disease, diabetes, and dementia [2]. High weight status before pregnancy is associated with increased risk of maternal morbidity or mortality, and higher rates of obstetric complications [3]. Adverse offspring outcomes related to high maternal weight include increased risk of congenital anomalies, macrosomia, and stillbirth [3]. Typically, 70–90% of reproductive-aged women from high-income countries participate in the workforce [4,5,6]. Work conditions may add to or alleviate the progression of chronic disease and weight status [7]. Accordingly, the workplace has been identified by the World Health Organization (WHO) and the International Labor Organization as a key setting for improving employee health and wellbeing [8]. 

Given the role that many employers play in facilitating health access and insurance [8,9], employers have a vested interest in championing the health of female employees. Employer interest in obesity prevention is increasing, with the aim of lowering medical and productivity-related costs and improving employee wellbeing [10]. Annual obesity-related costs for full-time U.S. employees are estimated to exceed $73 billion (USD) [11], with higher medical expenditure and absenteeism costs for women due to obesity compared to those of men [11]. Workplaces have a legal and ethical imperative to safeguard employee wellbeing, whilst also addressing inherent gender and health inequities [8]. Voluntary investment in employee health and welfare, exceeding minimum legal requirements, could be considered ‘corporate social responsibility’. Looking after employees in this way has been linked to improved corporate competitiveness and productivity [8]. Given the number of women in the workplace [4,6] and the potential benefits to the employer, the aim of this paper is to highlight the workplace as a key public health platform to promote healthy lifestyles and reduce the burden of weight gain for preconception, pregnant, and postpartum women.

## 2. The Burden of Weight Gain in Preconception, Pregnant, and Postpartum Women 

Approximately 50% of women enter pregnancy with above an optimal preconception body mass index (BMI) [12]. Further, an estimated 50% of pregnant women gain weight exceeding the U.S. Institute of Medicine (IOM) recommendations for weight gain during pregnancy [13]. This excess weight gain is strongly predictive of postpartum weight retention and long-term higher weight status [14]. Indeed, women who exceed IOM recommendations have a 300% increased risk of obesity within two decades [14], and 40% of women entering pregnancy with an overweight BMI move into the obese BMI category within 12 months of giving birth [15]. This pattern is similar across high-income nations [3]. The postpartum period is a critical preconception phase, given that 70% of first-time mothers go on to have a subsequent pregnancy [16]. Children born to mothers with high weight status are more likely to be affected by overweight or obesity as they grow [13,15]. These generational factors contribute to a cycle of overweight, obesity, and increased health risk with measurable adverse health impacts for offspring and women across the preconception, pregnancy, and postpartum periods. Consequently, prevention of weight gain over this period is essential. 

## 3. Current Strategies to Address Weight Gain in Preconception, Pregnant, and Postpartum Women

Strong clinical trial evidence exists for the efficacy of lifestyle interventions to impact gestational weight gain during pregnancy. A meta-analysis of individual participant data (over 12,500 women) evaluating the efficacy of diet and physical activity interventions to reduce gestational weight gain demonstrated that such interventions decreased the likelihood of cesarean section and limited weight gain during pregnancy, on average, by 0.7 kg [17]. Research shows that even a small reduction in gestational weight gain can have a significant public health impact by reducing pregnancy-related complications such as pre-eclampsia, gestational diabetes, and preterm delivery [18]. A recent randomized controlled trial indicated that a six-month preconception lifestyle intervention for women with obesity and infertility resulted in a significantly lower energy intake long-term, compared to the control group [19]. Postpartum lifestyle interventions have also shown efficacy for weight loss and weight gain prevention [20]. These benefits are not without limitations; for example, the short duration of pregnancy may make it difficult to change habits and develop positive lifestyle behaviors [16]. Further, efforts to prevent weight gain during the antenatal period are conflated with the high proportion of women entering the healthcare system during pregnancy with overweight or obesity [3]. Ideally, long-term behavior change would commence preconception or inter-pregnancy and continue across the pregnancy and postpartum periods. Although efforts to improve weight outcomes for women of reproductive age are promising, clinical resources are insufficient to meet the contextual demands of this population. Antenatal care targeting health behaviors is generally only offered to women with higher weight, therefore missing opportunities for weight gain prevention and highlighting the gaps in current clinical practice guidelines [17]. Longer-term postpartum care often concentrates on child development [14] and psychosocial wellbeing [21], without a focus on weight management or healthy lifestyle behaviors [14]. Additionally, half of all pregnancies are unplanned, and many women do not typically identify with the preconception period as a distinct life stage necessitating lifestyle adjustment [14]. Health care messages and support must extend to key community platforms, including workplaces, and thus meet the diverse needs of women across all stages of life. Despite calls to design and implement health promotion programs for preconception, pregnant, and postpartum women within the workplace [9,22], to our knowledge, there are no workplace health promotion interventions focusing on improving lifestyles and preventing weight gain in this population.

## 4. Benefits of Workplace Health Promotion 

As a ‘contact point’ for wellbeing [8], the workplace may enable and empower women to make the best possible health decisions with consequent benefits including better dietary, physical activity and weight outcomes [23], with due regard for the cultivation of gender and health equity. This ‘contact point’ could provide crucial access to health promotion for preconception women, including women not planning a pregnancy, and for women not in frequent contact with a healthcare setting. Furthermore, this approach acknowledges the possibility of any preconception woman, at any given time, to be undergoing a ‘pregnancy experience’, that is a pregnancy, a miscarriage or a stillbirth [24]. Explorative qualitative studies are essential to understand the scope of the individual needs specific to working women. Consequently, the distinct and, potentially, discreet needs of these women must be met with organizational support and incorporated seamlessly within a workplace setting. Healthy working environments could also benefit women with families by incorporating flexible work arrangements and assisting with the back-to-work transition.

Improvements to the work environment complement health behavior changes by influencing the intersection between work, leisure, and family life and yet, to date, the primary focus of employers has been to improve individual lifestyle behaviors alone [8]. Conflict between the work and home (e.g., work demands are prioritized over personal or family life) has been linked to worsened dietary behaviors [7]. Working at low control (a lack of autonomy over work tasks and schedules) and low reward workplaces, which typically employ women, is associated with poorer physical and mental health [21]. For example, shiftwork increases the risk of miscarriage, heart disease, breast cancer [8], and of postpartum depression in new mothers [21]. Work scheduling factors contribute to obesity, reduced physical activity, and the development of chronic disease [7]. A systems perspective, built on behavioral and environmental context, recognizes the intersectional relationship between the health of a business, its workforce, and the wider community [8]. In demonstrating dedication to employee wellbeing through provision of workplace health promotion, the employee-employer relationship is strengthened [25] and could also address social determinants of health such as income, job control or autonomy [26]. Potential for impact is apparent: young adult employees exposed to healthy food environments in the workplace are significantly less likely to have obesity [23]. Although tailored workplace health behavior interventions targeting weight loss have shown promise for women [27], research specific to women of reproductive age is absent from the literature. Provision of healthy food options at meetings and limiting alcohol-centered workplace engagements would be a simple way to support women who may be pregnant or planning a pregnancy to meet their health and weight goals, whilst maintaining their preferred level of discretion. Furthermore, workplaces that focus on addressing women’s needs throughout the preconception, pregnancy and postpartum periods may reap the added benefit of improving general employee wellbeing while focusing on the specific and unique needs of women through their reproductive life phase.

## 5. Barriers to Workplace Health Promotion 

Several barriers, both in and out of the workplace, may prevent women from engaging with workplace health promotion designed to prevent weight gain. As mentioned, there are no published studies focusing on the workplace barriers to lifestyle change specific to preconception, pregnant, and postpartum working women. However, general barriers include: heavy workloads, health promotion activity scheduling and location, work patterns such as part-time work or shift work, workplace location, limited availability of healthy lifestyle activities [28], and unsupportive management [29]. Additional obstacles impacting pregnant and postpartum women underscore the importance of tailored health promotion for this population, including health concerns such as gestational diabetes or morning sickness; short interconception periods, which may not be long enough to return to a healthy weight; and children’s health problems [30]. Targeting women during pregnancy and postpartum alone may mean that preconception women are excluded from potential health protection and prevention measures, which could include teratogen exposure around the time of conception or during the early stages of pregnancy [31]. Barriers could be addressed with workplace health promotion that promotes intervention uptake at multiple levels across the workplace organizational structure as well as the individual level for all women during the preconception, pregnancy and postpartum periods.

## 6. Tailoring Workplace Health Promotion for Preconception, Pregnant and Postpartum Women

Workplaces are diverse communities, built on organizational structures, psychosocial employee needs and behaviors, and the physical environment [7]. Consequently, a one-size-fits-all approach is not appropriate, and workplace health promotion interventions require solutions tailored to the specific workplace context and the needs of employees [7]. Intervention design frameworks, for example the Intervention Mapping [32] and Reach, Effectiveness, Adoption, Implementation, and Maintenance (RE-AIM; [33]) frameworks, may provide useful guides to facilitate this process through their use of participatory approaches and potential to feasibly achieve behavior change, mediated by employee self-efficacy and goal-setting [32]. The frameworks share commonalities including stakeholder engagement, evidence translation, and a focus on sustainability and effectiveness. Stakeholder involvement in program design and tailoring processes would address unique barriers to sustainability and facilitate buy-in [14]. Stakeholder engagement should continue across all aspects of intervention design including needs assessments, problem identification, co-development, testing, and evaluation [32]. Co-design processes should ensure that interventions are relevant and adaptable to the individual workplace system, convenient to access, and capable of scale-up. An example might be to co-develop an online portal, designed to integrate with existing workplace platforms and services, whilst providing discrete access and support to preconception, pregnant and postpartum women and alleviating potential health inequities or workplace discrimination. Such an intervention would also allow the possibility to include resources for team members, managers and human resources so that they can ensure they have the appropriate knowledge and skills to support women during this time. This could be considered particularly important for preconception women and women in the first trimester of pregnancy who may not be ready to share their private experiences within a public workspace [24].

In addition to individual employee behaviors, each workplace is influenced by the broader policy and regulatory activities within which the workplace exists. Consequently, interventions should also tailor to the specific workplace “system” [32], thus recognizing the impact of interrelated factors such as legal and political influences on employee health [7]. Additional government and business lobbying to influence policy and regulatory activities is an essential and constructive approach to health protection. Given that women constitute a disproportionate amount of the informal workforce (e.g., unpaid or casual) and do not benefit from the same level of legal and policy protections as permanent employees [8], it is also essential to address issues of gender inequality. Policies such as (in)adequate parental leave, increased care duties, and an “always on” approach to the modern workday disproportionately affect women and contribute to overall stress and work-family conflict [7]. Workplace stressors may also influence new mothers’ mental and physical health, which further emphasizes the need for health protective legislature and public health initiatives for women of reproductive age [21]. 

## 7. Conclusion

Women are increasingly entering pregnancy with a high BMI and are gaining weight across the reproductive life phase. Given the large number of reproductive-aged working women, the workplace is a key setting for improving the lifestyle, health, and weight status of women during the preconception, pregnancy and postpartum periods. While benefits to workplaces include improved economic, health and employee satisfaction outcomes, potential barriers to implementing workplace health promotion activities include limited activity availability and scheduling. Stakeholder consultation and co-design processes for interventions can be used to address these barriers, and improvements to broader policy and regulatory activities may also be beneficial. We need to focus on workplaces as an important, pragmatic setting to mitigate the public health problem of poor lifestyles, overweight and obesity across the preconception, pregnancy and postpartum periods. 
